# Supplementary material for: Genetic variation for fusarium crown rot tolerance in durum wheat
Source: PLoS One. 2021 Feb 12;16(2):e0240766. doi: 10.1371/journal.pone.0240766 (PMC7880437; doi:10.1371/journal.pone.0240766)
Supplement: S1 File — (DOCX) [file pone.0240766.s001.docx]

**Supplementary Material**

﻿**S.1** **Analysis of CRI**

The following scripts were used for the analysis of the 2017 CRI data. The 2016 analysis was conducted similarly.

require(ASExtras4)

require(asreml)

cri17.asrnull <- asreml(y^(2/3) ~ CR,

random =~ Genotype + Genotype:CR + Block + Mainplot,

residual = ~ar1(Column):ar1(Row),

na.action = na.method(x='include'), data=cri17.df, workspace=6e8)

# CORGH:

cri17.asr <- asreml((y)^(2/3) ~ CR,

random =~ corgh(CR):Genotype + Block + Mainplot,

residual = ~ar1(Column):ar1(Row),

na.action = na.method(x='include'), data=cri17.df, workspace=6e8)

lrt(cri17.asr,cri17.asrnull)

# now test all of the three components

# corr and minus variance and plus variance

# shows clearly only variance in plus

testrho17.asr <- asreml((y)^(2/3) ~ CR,

random =~ at(CR,'-'):Genotype + at(CR,'+'):Genotype +

Block + Mainplot,

residual = ~ar1(Column):ar1(Row),

na.action = na.method(x='include'), data=cri17.df, workspace=6e8)

lrt(cri17.asr,testrho17.asr)# correlation p-value = 0.1033

testm17.asr <- asreml((y)^(2/3) ~ CR,

random =~ at(CR,'+'):Genotype - at(CR,'-'):Genotype + Block + Mainplot,

residual = ~ar1(Column):ar1(Row),

na.action = na.method(x='include'), data=cri17.df, workspace=6e8)

lrt(testrho17.asr,testm17.asr) # minus variance p-value = 0.08276

testp17.asr <- asreml((y)^(2/3) ~ CR,

random =~ at(CR,'-'):Genotype - at(CR,'+'):Genotype +

Block + Mainplot,

residual = ~ar1(Column):ar1(Row),

na.action = na.method(x='include'), data=cri17.df, workspace=6e8)

lrt(testrho17.asr,testp17.asr) # plus variance p-value = 0.00575 **

cri17.pvs <- predict(cri17.asr,classify = 'Genotype:CR')

cri17.pred <- data.frame(Genotype=levels(cri17.df$Genotype))

cri17.pred$CRm <- subset(cri17.pvs$pvals,CR=='-')$predicted.value

cri17.pred$CRp <- subset(cri17.pvs$pvals,CR=='+')$predicted.value

**S.2 Analysis of grain yield**

The following scripts were used for the analysis of the grain yield data:

require(asreml)

asrat <- asreml(yield ~ CR*Trial,

random =~ at(Trial):Genotype:corgh(CR) - diag(Trial):Genotype:cor(CR)+ at(Trial):Block + at(Trial):Block:Mainplot + at(Trial, rrow):Row,

residual = ~dsum(~ar1(Column):ar1(Row)| Trial, levels = aa) +

dsum(~id(Column):ar1(Row) | Trial, levels = ia),

na.action = na.method(x='include'), data=cr1518.df, workspace=6e8)

test.asrat <- asreml(yield ~ CR*Trial,

random =~ at(Trial):Genotype:rr(CR) + at(EnvFCRTrt,c(2,3,5,8)):Genotype + at(Trial):Block + at(Trial):Block:Mainplot + at(Trial, rrow):Row,

residual = ~dsum(~ar1(Column):ar1(Row)| Trial, levels = aa) + dsum(~id(Column):ar1(Row) | Trial, levels = ia),

na.action = na.method(x='include'), data=cr1518.df, workspace=6e8)

##################

# test the presence of some tolerance across all years

# using rr() + atEnvFCRTrt) model which chooses which CR trt is larger variance

testall.asrat <- asreml(yield ~ CR*Trial,

random =~ at(Trial):Genotype:rr(CR) +

at(Trial):Block + at(Trial):Block:Mainplot +

at(Trial, rrow):Row,

residual = ~dsum(~ar1(Column):ar1(Row)| Trial, levels = aa) +

dsum(~id(Column):ar1(Row) | Trial, levels = ia),

na.action = na.method(x='include'), data=cr1518.df, workspace=6e8)

lrt(test.asrat,testall.asrat) # p-value = 8.637e-12 ***

cr1518.df$EnvFCRTrt <- factor(paste(cr1518.df$Trial,cr1518.df$CR,sep=':'))

##################

# Non-Separable FA(1)

asrtrcrW1 <- asreml(yield ~ Trial*CR,

random =~ fa(EnvFCRTrt,1):Genotype +

at(Trial):Block + at(Trial):Block:Mainplot + at(Trial, rrow):Row,

residual = ~dsum(~ar1(Column):ar1(Row)| Trial, levels = aa) +

dsum(~id(Column):ar1(Row) | Trial, levels = ia),

na.action = na.method(x='include'), data=cr1518.df, workspace=6e8)

# Non-Separable FA(2):

asrtrcrW2 <- asreml(yield ~ Trial*CR,

random =~ fa(EnvFCRTrt,2):Genotype +

at(Trial):Block + at(Trial):Block:Mainplot + at(Trial, rrow):Row,

residual = ~dsum(~ar1(Column):ar1(Row)| Trial, levels = aa) +

dsum(~id(Column):ar1(Row) | Trial, levels = ia),

na.action = na.method(x='include'), data=cr1518.df, workspace=6e8)

# Non-Separable FA(3):

asrtrcr <- asreml(yield ~ Trial*CR,

random =~ fa(EnvFCRTrt,3):Genotype +

at(Trial):Block + at(Trial):Block:Mainplot + at(Trial, rrow):Row,

residual = ~dsum(~ar1(Column):ar1(Row)| Trial, levels = aa) +

dsum(~id(Column):ar1(Row) | Trial, levels = ia),

na.action = na.method(x='include'), data=cr1518.df, workspace=6e8)

vv <- matrix((summary(asrtrcr,vparameters=T)$vparameters[[1]]),ncol=4)

gmat <- vv[,2:4]%*%t(vv[,2:4]) + diag(vv[,1])

Lam <- vv[,2:4]

Psi <- diag(vv[,1])

##################

# fit of the asrcorgh model and then try to fit the fa1 model

# Separable CORGH:

sv <- asreml(yield ~ CR*Trial,

random =~ corgh(Trial):Genotype:corgh(CR) +

at(Trial):Block + at(Trial):Block:Mainplot +

at(Trial, rrow):Row,

residual = ~dsum(~ar1(Column):ar1(Row)| Trial, levels = aa) +

dsum(~id(Column):ar1(Row) | Trial, levels = ia), start.values = TRUE,

na.action = na.method(x='include'), data=cr1518.df, workspace=6e8)

sv$vparameters.table[12,'Value'] <- .1

sv$vparameters.table[12,'Constraint'] <- 'F'

asrcorghW <- asreml(yield ~ CR*Trial,

random =~ corgh(Trial):Genotype:corgh(CR) +

at(Trial):Block + at(Trial):Block:Mainplot + at(Trial, rrow):Row,

G.param = sv$vparameters.table,

residual = ~dsum(~ar1(Column):ar1(Row)| Trial, levels = aa) +

dsum(~id(Column):ar1(Row) | Trial, levels = ia),

na.action = na.method(x='include'), data=cr1518.df, workspace=6e8)

asrcorgh <- asreml(yield ~ CR*Trial,

random =~ corgh(Trial):Genotype:cor(CR) +

at(Trial):Block + at(Trial):Block:Mainplot + at(Trial, rrow):Row,

residual = ~dsum(~ar1(Column):ar1(Row)| Trial, levels = aa) +

dsum(~id(Column):ar1(Row) | Trial, levels = ia),

na.action = na.method(x='include'), data=cr1518.df, workspace=6e8)

# Separable FA(1):

sv <- asreml(yield ~ CR*Trial,

random =~ fa(Trial):Genotype:corgh(CR) +

at(Trial):Block + at(Trial):Block:Mainplot + at(Trial, rrow):Row,

residual = ~dsum(~ar1(Column):ar1(Row)| Trial, levels = aa) +

dsum(~id(Column):ar1(Row) | Trial, levels = ia), start.values = TRUE,

na.action = na.method(x='include'), data=cr1518.df, workspace=6e8)

sv$vparameters.table[10,'Value'] <- .1

sv$vparameters.table[10,'Constraint'] <- 'F'

asrfa1W <- asreml(yield ~ CR*Trial,

random =~ fa(Trial):Genotype:corgh(CR) +

at(Trial):Block + at(Trial):Block:Mainplot + at(Trial, rrow):Row,

G.param = sv$vparameters.table,

residual = ~dsum(~ar1(Column):ar1(Row)| Trial, levels = aa) +

dsum(~id(Column):ar1(Row) | Trial, levels = ia),

na.action = na.method(x='include'), data=cr1518.df, workspace=6e8)

asrfa1 <- asreml(yield ~ CR*Trial,

random =~ fa(Trial):Genotype:cor(CR) +

at(Trial):Block + at(Trial):Block:Mainplot + at(Trial, rrow):Row,

residual = ~dsum(~ar1(Column):ar1(Row)| Trial, levels = aa) +

dsum(~id(Column):ar1(Row) | Trial, levels = ia),

na.action = na.method(x='include'), data=cr1518.df, workspace=6e8)

c(summary(asrtrcr)$aic,summary(asrcorghW)$aic,summary(asrfa1W)$aic)

# [1] 1] -757.4504 -748.3751 -732.6222 hence fa(3) better

eecorgh <- summary(asrcorgh,vparameters=T)$vparameters[[1]]

tempasr <- asreml(yield ~ CR*Trial,

random =~ corgh(Trial):Genotype:cor(CR) +

at(Trial):Block + at(Trial):Block:Mainplot - at(Trial, rrow):Row,

residual = ~dsum(~ar1(Column):ar1(Row)| Trial, levels = aa) +

dsum(~id(Column):ar1(Row) | Trial, levels = ia),

na.action = na.method(x='include'), data=cr1518.df, workspace=6e8)

lrt(tempasr,asrcorgh) # p = .007156 retain rrow

##################

# predictions

# and this checks with the coef

# need to NA those which are not present in the data

# need a data frame of the

# CR(+,-) for each geno x trial

# then need extra column of the difference between + and -

# for each year and then the average across years

# refit to get means rather than BLUPS

# change fixed effect to match EnvFCRTrt

# not Trial*CR for AIC

asrtrcr.final <- asreml(yield ~ EnvFCRTrt,

random =~ fa(EnvFCRTrt,3):Genotype +

at(Trial):Block + at(Trial):Block:Mainplot + at(Trial, rrow):Row,

residual = ~dsum(~ar1(Column):ar1(Row)| Trial, levels = aa) +

dsum(~id(Column):ar1(Row) | Trial, levels = ia),

na.action = na.method(x='include'), data=cr1518.df, workspace=6e8,maxit=13)

asrtrcr.finalrr <- asreml(yield ~ EnvFCRTrt,

random =~ rr(EnvFCRTrt,3):Genotype + diag(EnvFCRTrt):Genotype +

at(Trial):Block + at(Trial):Block:Mainplot +

at(Trial, rrow):Row,

residual = ~dsum(~ar1(Column):ar1(Row)| Trial, levels = aa) +

dsum(~id(Column):ar1(Row) | Trial, levels = ia),

na.action = na.method(x='include'), data=cr1518.df, workspace=6e8,maxit=13)

pvs <- predict(asrtrcr.final,classify = 'EnvFCRTrt:Genotype',

only = "fa(EnvFCRTrt, 3):Genotype", vcov = T)

nrow(pvs$pvals) # 288 = 4 x 36 x 2

##################

# now first form the tolerance index

# which is yield(+) - yield(-)

# predictions are ordered

# Trial/CR/Geno

# hence need three matrices

# form Lam and Psi for tol

Dtols <- kronecker(diag(4),matrix(c(-1,1),1,2))

tol.gmat <- Dtols%*%gmat%*%t(Dtols)

cov2cor(tol.gmat) # some agreement ranging from 0.43 to 0.62

(tolLam <- Dtols%*%Lam)

(tolPsi <- Dtols%*%Psi%*%t(Dtols))

mean(diag(tolPsi)/diag(tol.gmat))

ge.pres <- with(cr1518.df,table(Genotype,Trial))

ng <- length(levels(cr1518.df$Genotype))

Dtol <- kronecker(kronecker(diag(4),matrix(c(-1,1),1,2)),diag(ng))

tol.blup <- Dtol%*%pvs$pvals$predicted.value

Dminus <- kronecker(kronecker(diag(4),matrix(c(1,0),1,2)),diag(ng))

minus.blup <- Dminus%*%pvs$pvals$predicted.value

Dplus <- kronecker(kronecker(diag(4),matrix(c(0,1),1,2)),diag(ng))

plus.blup <- Dplus%*%pvs$pvals$predicted.value

Dminuss <- kronecker(diag(4),matrix(c(1,0),1,2))

minusLam <- Dminuss%*%Lam

minusPsi <- Dminuss%*%Psi%*%t(Dminuss)

Dboths <- kronecker(diag(4),matrix(c(-1,1,1,0),2,2,byrow=T))

bothLam <- Dboths%*%Lam

bothPsi <- Dboths%*%Psi%*%t(Dminuss)

###############

# form vcov of Dtol

# order is Trial/Genotype

# 4/36

# so diag(4)otimes Diff where Diff is 35 by 36

tol.vcov <- Dtol%*%pvs$vcov%*%t(Dtol)

I35 <- diag(rep(1,35))

suntop.con <- cbind(I35[,1:18],-1,I35[,19:35])

suntop.con <- kronecker(diag(4),suntop.con)

dim(suntop.con) # 140 x 144 good

tol.tval <- suntop.con%*%tol.blup/ sqrt(diag(suntop.con%*%tol.vcov%*%t(suntop.con)))

tol.df <- expand.grid(Genotype=levels(cr1518.df$Genotype), Trial=levels(cr1518.df$Trial))

tol.df$pres <- as.vector(ge.pres)==0

tol.df$blup <- tol.blup

tol.df$blup[tol.df$pres] <- NA

tol.df$tval <- rep(NA,36*4)

tol.df$tval[tol.df$Genotype!='SUNTOP'] <- tol.tval

tol.df$tval[tol.df$pres] <- NA

tol.df$pval <- pnorm(tol.df$tval)

tol.df$LOD <- -log10(tol.df$pval)

temp.fdr <- subset(tol.df,!is.na(pval))

temp.fdr <- temp.fdr[order(temp.fdr$pval),]

qstar <- .01

temp.fdr$fdrcut <- (1:nrow(temp.fdr))*qstar/nrow(temp.fdr)

temp.fdr$significant01 <- with(temp.fdr, pval<fdrcut)

temp.fdr <- temp.fdr[order(temp.fdr$Trial,temp.fdr$Genotype),]

tol.df$sign01 <- rep(NA,36*4)

tol.df$sign01[!is.na(tol.df$tval)] <- temp.fdr$significant01

tolLOD.mat <- matrix(tol.df$LOD,ncol=4)

dimnames(tolLOD.mat) <- list(levels(cr1518.df$Genotype),levels(cr1518.df$Trial))

###############

# OP for TOL

# need to use mbf trick to get the PEV for the scores of the 8 levels

# by creating a factor with 8+fa(3) extra levels

mbflev <- levels(cr1518.df$ EnvFCRTrt)

cr1518.df$mbffac <- as.character(cr1518.df$ EnvFCRTrt)

cr1518.df$mbffac <- factor(cr1518.df$mbffac,levels=c(mbflev,'fac1','fac2','fac3'))

levels(cr1518.df$mbffac)

#[1] "15S4CRTOL:-" "15S4CRTOL:+" "16S4CRTOL:-" "16S4CRTOL:+" "17S4CRTOL:-" #[6] "17S4CRTOL:+" "18S4CRTOL:-" "18S4CRTOL:+" "fac1" "fac2" "fac3"

mbf.df <- as.data.frame(rbind(Lam,diag(3)))

mbf.df$mbffac <- levels(cr1518.df$mbffac)

# make sure asreml keeps levels in the data which are not there

asreml.options(workspace='2000mb', drop.unused.levels="FALSE")

sv <- asreml(yield ~ EnvFCRTrt,

random =~ idv(mbf(load)):Genotype + diag(EnvFCRTrt):Genotype +

at(Trial):Block + at(Trial):Block:Mainplot + at(Trial, rrow):Row,

residual = ~dsum(~ar1(Column):ar1(Row)| Trial, levels = aa) +

dsum(~id(Column):ar1(Row) | Trial, levels = ia),start.values = T,

mbf = list('load'=list(key=c('mbffac','mbffac'), cov = 'mbf.df')),

na.action = na.method(x='include'), data=cr1518.df)

# set variance for mbf to 1

mydiff <-list('mbf'=list('mbf'=1))

ee <- update.mfxlm(obj=asrtrcr.finalrr,sv=sv,diff=mydiff,boundfix = FALSE)

# to be on safe side fix all variances here as well as in call

ee$G.sv$Constraint <- 'F'

ee$R.sv$Constraint <- 'F'

asrmbf <- asreml(yield ~ EnvFCRTrt,

random =~ idv(mbf(load)):Genotype + diag(EnvFCRTrt):Genotype +

at(Trial):Block + at(Trial):Block:Mainplot + at(Trial, rrow):Row,

residual = ~dsum(~ar1(Column):ar1(Row)| Trial, levels = aa) +

dsum(~id(Column):ar1(Row) | Trial, levels = ia),R.param = ee$R.sv,

G.param = ee$G.sv, maxit=1,

mbf = list('load'=list(key=c('mbffac','mbffac'), cov = 'mbf.df')),

na.action = na.method(x='include'), data=cr1518.df)

####################

# now get predictions of factors and their variance

# have to look for interaction with Genotype

ff <- asrmbf$factor.names

(ff <- ff[grep('mbf',ff)])

(ff <- ff[grep('mbf.*:',ff)])

predlev.df <- data.frame(Genotype=rep(levels(cr1518.df$Genotype),3), mbffac=rep(c('fac1','fac2','fac3'), each=length(levels(cr1518.df$Genotype))))

# order prediction levels as varieties within factors

mbf.pvs <- predict(asrmbf, classify='mbffac:Genotype', only=ff,

maxit = 1, parallel = TRUE, levels=list('mbffac'=predlev.df$mbffac, 'Genotype'=predlev.df$Genotype), vcov=T)

###############

# get tolerance CVE blups

tol.cve <- kronecker(tolLam,diag(36))%*%mbf.pvs$pvals$predicted.value

plot(as.vector(matrix(cc,ncol=11)[,1:8]%*%t(Dtols)),tol.cve)

#################

# now rotate Lamtol

# to get rotated scores as well

ssl <- svd(tolLam)

V <- ssl$v

t(V)%*%V

V%*%t(V)

tolLamstar <- -tolLam%*%V

round(t(tolLamstar)%*%tolLamstar,3)

tol.f <- mbf.pvs$pvals$predicted.value

tol.fstar <- kronecker(-t(V),diag(36))%*%tol.f

###################

# final bit

I35 <- diag(rep(1,35))

suntop.con <- cbind(I35[,1:18],-1,I35[,19:35])

dim(suntop.con) # 35 x 36

PEV.f <- mbf.pvs$vcov

PEV.fstar <- kronecker(-t(V),diag(36))%*%PEV.f%*%

t(kronecker(-t(V),diag(36)))

tol.fstar <- tol.fstar[1:36]

PEV.fstar <- PEV.fstar[1:36,1:36]

sed.fstar <- sqrt(diag(suntop.con%*%as.matrix(PEV.fstar)%*%t(suntop.con)))

tol.fstar.tval <- suntop.con%*%tol.fstar/sed.fstar

tolfstar.df <- expand.grid(Genotype=levels(cr1518.df$Genotype))

tolfstar.df$tval <- rep(NA,36)

tolfstar.df$tval[tolfstar.df$Genotype!='SUNTOP'] <- tol.fstar.tval

tolfstar.df$pval <- pnorm(tolfstar.df$tval)

tolfstar.df$LOD <- -log10(tolfstar.df$pval)

tolfstar.df$fstar <- tol.fstar

tolfstar.df$PEVf <- diag(PEV.fstar)

tolfstar.df$fstarlower <- tolfstar.df$fstar - 1.96*sqrt(tolfstar.df$PEVf)

tolfstar.df$fstarupper <- tolfstar.df$fstar + 1.96*sqrt(tolfstar.df$PEVf)

temp.fdr <- subset(tolfstar.df,!is.na(pval))

temp.fdr <- temp.fdr[order(temp.fdr$pval),]

qstar <- .01

temp.fdr$fdrcut <- (1:nrow(temp.fdr))*qstar/nrow(temp.fdr)

temp.fdr$significant01 <- with(temp.fdr, pval<fdrcut)

temp.fdr <- temp.fdr[order(temp.fdr$Genotype),]

tolfstar.df$sign01 <- rep(NA,36)

tolfstar.df$sign01[!is.na(tolfstar.df$tval)] <- temp.fdr$significant01

# wrapup

# form the matrix of blups for tol, plus, minus

plus.df <- expand.grid(Genotype=levels(cr1518.df$Genotype),

Trial=levels(cr1518.df$Trial))

plus.df$pres <- as.vector(ge.pres)==0

plus.df$blup <- plus.blup

plus.df$blup[plus.df$pres] <- NA

minus.df <- expand.grid(Genotype=levels(cr1518.df$Genotype),

Trial=levels(cr1518.df$Trial))

minus.df$pres <- as.vector(ge.pres)==0

minus.df$blup <- minus.blup

minus.df$blup[minus.df$pres] <- NA

plot.df <- expand.grid(Genotype=levels(cr1518.df$Genotype))

plot.df <- cbind(plot.df,matrix(tol.df$blup,ncol=4), matrix(minus.df$blup,ncol=4),matrix(plus.df$blup,ncol=4))

names(plot.df)[-1] <- c('tol15','tol16','tol17','tol18','minus15', 'minus16','minus17','minus18','plus15','plus16','plus17','plus18')

**S.3 Tables and figures for the analysis of CRI and grain yield**

The following scripts were used to produce the tables and figures for the analysis of the CRI and the grain yield data:

# tables and figures

require(xtable)

val16 <- summary(cri16.asr)$varcomp[c('CR:Genotype!CR_-','CR:Genotype!CR_+','CR:Genotype!CR!+:!CR!-.cor'),]

ll16m <- lrt(testrho16.asr,testm16.asr)$`Pr(Chisq)`

ll16p <- lrt(testrho16.asr,testp16.asr)$`Pr(Chisq)`

ll16rho <- (1- pchisq(lrt(cri16.asr,testrho16.asr)$'LR-statistic',df=1))

val17 <- summary(cri17.asr)$varcomp[c('CR:Genotype!CR_-','CR:Genotype!CR_+','CR:Genotype!CR!+:!CR!-.cor'),]

ll17m <- lrt(testrho17.asr,testm17.asr)$`Pr(Chisq)`

ll17p <- lrt(testrho17.asr,testp17.asr)$`Pr(Chisq)`

ll17rho <- (1- pchisq(lrt(cri17.asr,testrho17.asr)$'LR-statistic',df=1)) # not bounded issue

xx <- data.frame(Parameter=c('var(n)','var(i)','corr(n,i)'), est16=val16[,'component'], pval16=c(ll16m,ll16p,ll16rho),est17=val17[,'component'], pval17=c(ll17m,ll17p,ll17rho))

xx <- xtable(xx,caption='Summary of REML estimates of the genetic variance parameters for the analysis of CRI for 2016 and 2017.', floating=TRUE, latex.environments='center',label = 'tab:trialsum')

align(xx) <- xalign(xx)

digits(xx) <- c(0,0,4,3,4,3)

addtorow <- list()

addtorow$pos <- list(0,0)

addtorow$command <- c("& \\multicolumn{2}{c}{2016} & \\multicolumn{2}{c}{2016}\\\\\n ", "Parameter & Estimate & p-value & Estimate & p-value\\\\\n")

print(xx,include.colnames=FALSE,add.to.row=addtorow,include.rownames=FALSE,floating=TRUE,latex.environments='center',scalebox=1.0)

###################

# 2017 plot for analysis of CRI:

require(ggplot2)

ggplot(data = cri17.pred, mapping = aes(x = CRm^(3/2), y = CRp^(3/2), label = Genotype)) + geom_text() + xlab('CRI(FCR-n)') + ylab('CRI(FCR-i)')

#################

# baseline yield

vv <- summary(asrat)$varcomp

vv <- matrix(vv[grep('Genotype',dimnames(vv)[[1]]),'component'],nrow=3)

vv <- vv[c(2,3,1),]

dimnames(vv) <- list(c('var(FCR-n)','var(FCR-i)','cor(FCR-n,FCR-i)'), c('est15','est16','est17','est18'))

xx <- xtable(vv,caption='Summary of REML estimates of the genetic variance parameters for the analysis of grain yield using the baseline model.',

floating=TRUE, latex.environments='center',label = 'tab:basey')

align(xx) <- xalign(xx)

digits(xx) <- c(0,4,4,4,4)

addtorow <- list()

addtorow$pos <- list(0,0)

addtorow$command <- c("& \\multicolumn{4}{c}{Estimate}\\\\\n ", "Parameter & 2015 & 2016 & 2017 & 2018 \\\\\n")

print(xx,include.colnames=FALSE,add.to.row=addtorow,include.rownames=TRUE, floating=TRUE,latex.environments='center',scalebox=1.0)

##################

# full yield model

aaic <- c(summary(asrtrcrW1)$aic,summary(asrtrcrW2)$aic,

summary(asrtrcr)$aic,summary(asrcorghW)$aic,summary(asrfa1W)$aic)

logl <- c(summary(asrtrcrW1)$logl,summary(asrtrcrW2)$logl,

summary(asrtrcr)$logl,summary(asrcorghW)$logl,summary(asrfa1W)$logl)

vvfa1 <- summary(asrfa1W)$varcomp

vvcorgh <- summary(asrcorghW)$varcomp

vvfa1ns <- summary(asrtrcrW1)$varcomp

vvfa2ns <- summary(asrtrcrW2)$varcomp

vvfa3ns <- summary(asrtrcr)$varcomp

npar <- c(dim(vvfa1[grep('Genotype',dimnames(vvfa1)[[1]]),])[1], dim(vvcorgh[grep('Genotype',dimnames(vvcorgh)[[1]]),])[1], dim(vvfa1ns[grep('Genotype',dimnames(vvfa1ns)[[1]]),])[1], dim(vvfa2ns[grep('Genotype',dimnames(vvfa2ns)[[1]]),])[1], dim(vvfa3ns[grep('Genotype',dimnames(vvfa3ns)[[1]]),])[1]) -1

xx <- data.frame(Type=c('Sep','Sep','Nonsep','Nonsep','Nonsep'), VModel=c('fa1(Env)$\\times $corgh-c(FCR)','corgh(Env)$\\times $corgh-c(FCR)', (EnvFCRTrt)','fa2(EnvFCRTrt)','fa3(EnvFCRTrt)'), npar=npar,logl=logl[c(5,4,1,2,3)], aic=aaic[c(5,4,1,2,3)])

xx$VModel <- as.character(xx$VModel)

xx <- xtable(xx,caption='Summary of model fits for grain yield 2015-2018: number of genetic variance parameters, REML log-likelihood and the AIC values', floating=TRUE, latex.environments='center',label = 'tab:models')

align(xx) <- xalign(xx)

digits(xx) <- c(0,0,0,0,1,1)

print(xx,include.colnames=TRUE,include.rownames=FALSE, sanitize.text.function = function(x) {x}, floating=TRUE, latex.environments='center',scalebox=1.0, type='latex')

##############

# now print the fitted gmat

ggm <- cov2cor(gmat)

summary(asrat)$varcomp

diag(ggm) <- diag(gmat)

dimnames(ggm) <- list(c('15-n','15-i','16-n','16-i','17-n','17-i','18-n','18-i'),('15-n','15-i','16-n','16-i','17-n','17-i','18-n','18-i'))

xx <- xtable(ggm,caption='REML estimate of the variance matrix for the concatenated factor \\texttt{EnvFCRTrt} using an FA3 non-separable model. The acronym corgh-c represents a constrained corgh variance model. Values on the upper and lower triangle are the estimated correlations between the effects for each level of \\texttt{EnvFCRTrt} and numbers on the diagonals are the estimated variances for each level of \\texttt{EnvFCRTrt}.', floating=TRUE, latex.environments='center', label = 'tab:models')

align(xx) <- xalign(xx)

digits(xx) <- c(0,rep(3,8))

print(xx,include.colnames=TRUE,include.rownames=TRUE, sanitize.text.function = function(x) {x}, floating=TRUE, latex.environments='center',scalebox=1.0, type='latex')

##############

# the table of FCR tolerance

xx <- xtable(tolfstar.df[order(-tolfstar.df$fstar),], caption='Summary of the FCR tolerance indices for each genotype, ordered on the standardised FCR index along with t-statistics, p-values, LOD scores and 95\\% coverage intervals for the standardised FCR index.',floating=TRUE, latex.environments='center',label = 'tab:fcrtol')

align(xx) <- xalign(xx)

digits(xx) <- c(0,0,2,4,2,0,3,3,3,3)

print(xx,include.colnames=TRUE,include.rownames=FALSE, sanitize.text.function = function(x) {x}, floating=TRUE, latex.environments='center',scalebox=1.0, type='latex')

#########

library(ggrepel)

# 2015

oo <- ggplot(data = plot.df,mapping = aes(x=minus15, y=tol15, label=Label, color=Label)) + geom_text_repel() + theme(legend.position = "none")

oo15 <- oo + xlab("Yield for FCR-n") + ylab("FCR Tolerance") + ggtitle("2015") + geom_point() + theme(legend.position = "none", aspect.ratio = 1, axis.text = element_text(size = 10), axis.title = element_text(size = 15, face = "bold"))

pp <- ggplot(data = plot.df,mapping = aes(x=minus15, y=plus15, label=Label, color=Label)) + geom_text_repel() + theme(legend.position = "none")

pp2015 <- pp + xlab("Yield for FCR-n") + ylab("Yield for FCR-i") + ggtitle("2015") + geom_point() + theme(legend.position = "none", aspect.ratio = 1, axis.text = element_text(size = 10), axis.title = element_text(size = 15, face = "bold"))

# 2016

oo <- ggplot(data = plot.df,mapping = aes(x=minus16, y=tol16, label=Label, color=Label)) + geom_text_repel() + theme(legend.position = "none")

oo16 <- oo + xlab("Yield for FCR-n") + ylab("FCR Tolerance") + ggtitle("2016") + geom_point() + theme(legend.position = "none", aspect.ratio = 1, axis.text = element_text(size = 10), axis.title = element_text(size = 15, face = "bold"))

pp <- ggplot(data = plot.df,mapping = aes(x=minus16, y=plus16, label=Label, color=Label)) + geom_text_repel() + theme(legend.position = "none")

pp2016 <- pp + xlab("Yield for FCR-n") + ylab("Yield for FCR-i") + ggtitle("2016") + geom_point() + theme(legend.position = "none", aspect.ratio = 1, axis.text = element_text(size = 10), axis.title = element_text(size = 15, face = "bold"))

# 2017

oo <- ggplot(data = plot.df,mapping = aes(x=minus17, y=tol17, label=Label, color=Label)) + geom_text_repel() + theme(legend.position = "none")

oo17 <- oo + xlab("Yield for FCR-n") + ylab("FCR Tolerance") + ggtitle("2017") + geom_point() + theme(legend.position = "none", aspect.ratio = 1, axis.text = element_text(size = 10), axis.title = element_text(size = 15, face = "bold"))

pp <- ggplot(data = plot.df,mapping = aes(x=minus17, y=plus17, label=Label, color=Label)) + geom_text_repel() + theme(legend.position = "none")

pp2017 <- pp + xlab("Yield for FCR-n") + ylab("Yield for FCR-i") + ggtitle("2017") + geom_point() + theme(legend.position = "none", aspect.ratio = 1, axis.text = element_text(size = 10), axis.title = element_text(size = 15, face = "bold"))

# 2018

oo <- ggplot(data = plot.df,mapping = aes(x=minus18, y=tol18, label=Label, color=Label)) + geom_text_repel() + theme(legend.position = "none")

oo18 <- oo + xlab("Yield for FCR-n") + ylab("FCR Tolerance") + ggtitle("2018") + geom_point() + theme(legend.position = "none", aspect.ratio = 1, axis.text = element_text(size = 10), axis.title = element_text(size = 15, face = "bold"))

pp <- ggplot(data = plot.df,mapping = aes(x=minus18, y=plus18, label=Label, color=Label)) + geom_text_repel() + theme(legend.position = "none")

pp2018 <- pp + xlab("Yield for FCR-n") + ylab("Yield for FCR-i") + ggtitle("2018") + geom_point() + theme(legend.position = "none", aspect.ratio = 1, axis.text = element_text(size = 10), axis.title = element_text(size = 15, face = "bold"))

library(gridExtra)

grid.arrange(pp2015, pp2016, pp2017, pp2018, nrow = 2)

grid.arrange(oo15, oo16, oo17, oo18, nrow = 2)

names(plot.df)[2:5] <- c("FCR Tolerance 2015","FCR Tolerance 2016","FCR Tolerance 2017","FCR Tolerance 2018")

bcplot1 <- ggplot(plot.df,aes(x=`FCR Tolerance 2015`, y=`FCR Tolerance 2016`, label=Label,color=Label)) + geom_text_repel(size=5) + geom_point() + theme(legend.position = "none", axis.title = element_text(size = 15, face = "bold"))

bcplot2 <- ggplot(plot.df, aes(x=`FCR Tolerance 2015`, y=`FCR Tolerance 2017`, label=Label,color=Label)) + geom_text_repel(size=5) + geom_point() + theme(legend.position = "none", axis.title = element_text(size = 15, face = "bold"))

bcplot3 <- ggplot(plot.df, aes(x=`FCR Tolerance 2015`, y=`FCR Tolerance 2018`, label=Label,color=Label)) + geom_text_repel(size=5) + geom_point() + theme(legend.position = "none", axis.title = element_text(size = 15, face = "bold"))

bcplot4 <- ggplot(plot.df, aes(x=`FCR Tolerance 2016`, y=`FCR Tolerance 2017`, label=Label,color=Label)) + geom_text_repel(size=5) + geom_point() + theme(legend.position = "none", axis.title = element_text(size = 15, face = "bold"))

bcplot5 <- ggplot(plot.df, aes(x=`FCR Tolerance 2016`, y=`FCR Tolerance 2018`, label=Label,color=Label)) + geom_text_repel(size=5) + geom_point() + theme(legend.position = "none", axis.title = element_text(size = 15, face = "bold"))

bcplot6 <- ggplot(plot.df, aes(x=`FCR Tolerance 2017`, y=`FCR Tolerance 2018`, label=Label,color=Label)) + geom_text_repel(size=5) + geom_point() + theme(legend.position = "none", axis.title = element_text(size = 15, face = "bold"))

grid.arrange(bcplot1,bcplot2,bcplot3,bcplot4,bcplot5,bcplot6,nrow=2)
