# Supplementary material for: Genetic variation for fusarium crown rot tolerance in durum wheat
Source: PLoS One. 2021 Feb 12;16(2):e0240766. doi: 10.1371/journal.pone.0240766 (PMC7880437; doi:10.1371/journal.pone.0240766)
Supplement: Attachment — Submitted filename: Kadkol et al Response to reviewers.docx [file pone.0240766.s002.docx]

**Response to Reviewer**

**PONE-D-20-09326 - “Genetic variance for Fusarium crown rot tolerance in durum wheat”**

We thank the reviewer for his kind comments on our paper and helpful suggestions. We have accepted most of their suggestions and provided valid reasons where we didn’t accept their suggestions.

1. Line 16: Please change “checks” with “controls”;
   - Agreed. “checks” changed to “controls”
2. Line 39: Please be more precise and avoid the word “overseas”;
   - Agreed. The sentence has been changed to “…..is an important disease of cereals in Australia and other countries and regions, such as, USA, South Africa, North Africa, Italy, Middle East and China (Burgess et al. 2001; Smiley et al. 2005; Alahmad et al, 2018, Simpfendorfer et al. 2019).”
3. Line 41: Please indicate what mean “NSW”;
   - Sentence changed to “…………..production in northern New South Wales (NSW) and southern Queensland……………..”
4. Line 49: The author mention that there are no effective fungicide products available for management of FCR but maybe should be mentioned seed dressing;
   - We have amended the original sentence to “Currently, there are no effective seed or foliar fungicide products available for the management of FCR (Alahmad et al. 2018)”.
   - As discussed by Alahmad et al. (2018) the various chemical treatments tried either as post-emergent sprays or as seed treatments have shown limited or no efficacy for FCR control under field conditions. Currently, registered seed treatments in Australia are only registered for suppression of Fusarium crown rot which have limited activity. Seed treatments are really only for limiting seedling blight when sowing Fusarium head blight infected grain. This does not provide significant protection from infection coming from infected stubble during the season, which is the main pathway with Fusarium crown rot infection.
5. Lines 125-126: What is meant for “current elite Australian material”? Please specify;
   - Sentence amended to “…………..current elite Australian durum breeding material in the DBA program.”
6. Lines 126-128: This sentence is not clear;

- See below

7. Lines 125-131: To my opinion insert in the aim of the research, other additional comment could be not useful for the reader. I suggest to reformulate all these sentences;
   - Sentences amended to “Previous studies of disease tolerance have used several levels of disease pressure to determine tolerance in intensive tests of a small number of genotypes (Raberg et al. 2009; Forknall et al. 2019). However, this approach is not suitable for estimating genetic variation in a breeding setting because a higher number of genotypes need to be tested without making the experiments too large and cost prohibitive”.

8) Line 139-141: Please avoid the general use of the word “preliminary”, I suggest to be more precise in describe these preliminary trials;

- We would like to retain the original sentence which describes the nature of the work conducted prior to 2015. These trials were used to determine an optimum protocol for the 2015-17 trials that are presented in the manuscript and they were preliminary in nature.

9) Line 156 and lines 164-165: You don’t have the same genotypes each year. Some genotypes were tested only for one year and some other for two years and some others again for three years. Additional promising lines were added every year. Please describe better the reasons of this choice;

- Our reasoning was that it should be possible to screen for FCR tolerance using experiments with only an inoculated FCR treatment. We have demonstrated this by adding putatively better tolerant lines from other FCR treated trials into this study. We have discussed this in the Discussion section (lines 667-671).
- The text has been amended to “Some lines judged to be FCR-intolerant were replaced with new lines that were selected based on their performance in other FCR treated trials in the DBA North breeding program to improve the chances of identifying FCR-tolerant lines. However, a fairly high degree of concurrence between trials was maintained and this allowed the MET analysis of the data as discussed below”.

10) Line 172: What type of isolates did you use? Please be more precise about name, origin, host from which were obtained, etc.;

- We have added the following text and table: “ Although FCR isolates have been shown to vary in aggressiveness, there is no race structure established that causes differential varietal reactions (Akinsanmi et al. 2004). The details of the five isolates has been added as below: All isolates were established from hyphal tip cultures to ensure purity and identified by qPCR to be *Fusarium pseudograminearum* by SARDI*.* Isolates were collected from different commercial wheat crops with severe basal browning characteristic of Fusarium crown rot infection at harvest in either 2013 or 2014”.
- Table 3 Details of *Fp* isolates used in preparation of CR inoculum

| SARDI ID | Isolate ID | Year | State | Location | Host | qPCR ID |
| --- | --- | --- | --- | --- | --- | --- |
| 4093 AC29312 | CAS-13/94C | 2013 | NSW | Walgett | Wheat | *F. pseudograminearum* |
| 4093 AC29363 | CAS-13/131C | 2013 | NSW | Rowena | Wheat | *F. pseudograminearum* |
| 4095 AC29475 | CAS-13/161N | 2013 | NSW | Moree | Durum | *F. pseudograminearum* |
| 4849 BA48166 | CAS-14/98C | 2014 | NSW | Warren | Wheat | *F. pseudograminearum* |
| 4719 BA40558 | CAS 14/88N | 2014 | Qld | Moonie | Wheat | *F. pseudograminearum* |

11) Lines 169-174: Please describe better the inoculation technique on experimental plots, provide more details;

- Inoculation method is described in lines 152-153. “Plots inoculated with FCR were sown with inoculum mixed with viable seed at a rate of 2 g *Fp* inoculum/m row, as described by Dodman and Wildermuth (1987).” Full details of inoculum production and inoculation are outlined in Forknall et al. 2019

12) Line 542: DISCUSSION SECTION: I suggest to revise the entire section taking care to expand the results of this study in a wider research context. In the present form there are many indication about this study but a low level of citations of other studies that could incorporate this research in a more comprehensive research scenario;

- We wish to retain most of the original text but have incorporated the reviewer’s specific suggestions (13-15) because:
  - As we have explained, FCR tolerance is a new concept and there is little work reported in this area of study as reviewed by Forknall et al. (2019).
  - There have been no published reports of genetic variance in FCR tolerance in either bread wheat or durum wheat (except for reported tolerance in Caparoi by Forknall et al., 2019).
  - This study is the first demonstration of variation for FCR tolerance in durum wheat.

13) Lines 547-549: This sentence is not clear, please reformulate it;

- The sentence has been amended to “These differences were observed in all four seasons, including 2016, which resulted in low levels of CRI due to high rainfall in spring and favourable moisture conditions through the growing season (Figs 1 and 2) and this was consistent with the observations of Hollaway et al. (2013)”.
- Also, an additional graph of 2016 CRI data has been added to Fig 2 to support the comments.

14) Lines 568-569: I suggest to avoid the use of this sentence;

- Agreed. Sentence deleted.

15) Lines 611-612: The authors mention an uncertain relationship of the previous studies with field conditions, please be more accurate about this statement;

- “uncertain” amended to “unspecified”

16) Lines 622-624: This assertion is of crucial importance. Please expand it possibly adding some other studies (with references) were the difficult to combine high yield potential and disease tolerance were found;

- Thanks for this comment. We have amended the paragraph and added discussion on the likelihood of FCR tolerance have a yield penalty. We have also moved this paragraph towards the end of the discussion for better logical flow.
- Text has amended to “In this study FCR tolerance was strongly negatively correlated with yield predictions for FCR-n treatments in 2017, and moderately negatively related in 2015 and 2016 seasons (Fig 5). There were also significantly positive correlations observed between FCR tolerance values and yield from FCR-n treatments across the years (Table 9) and thus the overall correlation was moderately negative suggesting that it could be somewhat difficult to combine high yield potential under low disease or disease-free conditions and FCR tolerance. There are some examples, such as resistance from the *mlo* gene to barley powdery mildew (*Blumeria graminis* [syn. *Erysiphe graminis*] f. sp. *Hordei*) (Brown and Rant, 2013) and durable resistance to wheat leaf rust from the *Lr34* gene are associated with a yield penalty (Singh and Huerta-Espino, 1997). In a recent publication (Huerta-Espino et al. 2020), the findings of the yield penalty have been revised and the authors have noted that *LR34* together with other durable resistance genes forms the backbone of the CIMMYT bread wheat germplasm. However, in FCR, tolerance is even less likely to result in yield penalty because, as commented above, tolerance could be considered to result from drought tolerance traits, such as, optimum maturity, better root architecture, stay green, leaf rolling etc. which are all yield positive traits. Good FCR tolerance is required for high yields under Australian conditions because a large portion of cereal crops surveyed in recent years are reported to contain significant levels of FCR infection (Simpfendorfer et. al. 2019). Also, one of the genotypes identified as relatively tolerant in this study, DBA Bindaroi, has been a good performer for grain yield and grain quality in the Australian ACAS/NVT trials (<https://www.grdc-nvt.com.au/>) and it was released for commercial cultivation in 2017..”

17) Lines 626-629: This sentence could be important but I think they need of a reference;

- Reference (website address) added.

18) Lines 631-632: What pathosystem? Please be more accurate;

- Sentence amended to “The concept of tolerance is relatively new in FCR research, first demonstrated by Forknall et al. (2019) in bread wheat but is well known in other pathosystems, such as, blackleg disease of canola (Salisbury et al. 1995; Raman et al. 2013).”

We have also taken this opportunity to replace “line” with “genotype” for better consistency.

We have also made other corrections including a small change to the title of the paper. We trust this is okay.

We thank the referee for his time and constructive comments.

Gururaj Kadkol

(on behalf of all co-authors)
